# Effect of Cold Atmospheric Plasma Therapy vs Standard Therapy Placebo on Wound Healing in Patients With Diabetic Foot Ulcers: A Randomized Clinical Trial

**DOI:** 10.1001/jamanetworkopen.2020.10411

**Published:** 2020-07-16

**Authors:** Bernd Stratmann, Tania-Cristina Costea, Catharina Nolte, Jonas Hiller, Jörn Schmidt, Jörg Reindel, Kai Masur, Wolfgang Motz, Jürgen Timm, Wolfgang Kerner, Diethelm Tschoepe

**Affiliations:** 1 Diabeteszentrum, Herz- und Diabeteszentrum Nordrhein Westfalen (NRW), Ruhr Universität Bochum, Bad Oeynhausen, Germany; 2 Klinikum Karlsburg der Klinikgruppe Dr Guth GmbH & Co KG, Karlsburg, Germany; 3 Leibniz-Institut für Plasmaforschung und Technologie, Greifswald, Germany; 4 Kompetenzzentrum Diabetes Karlsburg, Karlsburg, Germany; 5 Competence Center for Clinical Studies Bremen, Bremen, Germany

## Abstract

**Importance:**

Diabetic foot ulcers are a common complication of diabetes and require specialized treatment. Cold atmospheric plasma (CAP) has been associated with benefits in wound infection and healing in previous smaller series of case reports. Yet the effect of CAP compared with standard care therapy in wound healing in diabetic foot ulcers remains to be studied.

**Objective:**

To determine whether the application of CAP accelerates wound healing in diabetic foot ulcers compared with standard care therapy.

**Design, Setting, and Participants:**

A prospective, randomized, placebo-controlled, patient-blinded clinical trial was conducted at 2 clinics with recruitment from August 17, 2016, to April 20, 2019. Patients were scheduled to remain in follow-up until April 30, 2024. Patients with diabetes and diabetic foot ulcers described using the combined Wagner-Armstrong classification of 1B or 2B (superficial or infected diabetic foot ulcers extending to tendon) were eligible. A patient could participate with 1 or more wounds in both groups in both intervention and control groups. Wounds were randomized separately, allowing a participant to be treated several times within the study following a 2 × 2 × 2 randomization strata considering sex, smoking status, and age (≤68 years and >68 years).

**Interventions:**

Standard care treatment with 8 applications of either CAP generated from argon gas in an atmospheric pressure plasma jet or 8 applications of placebo treatment in a patient-blinded manner.

**Main Outcomes and Measures:**

Primary end points were reduction in wound size, clinical infection, and microbial load compared with treatment start. Secondary end points were time to relevant wound reduction (>10%), reduction of infection, parameters of patient’s well-being, and treatment-associated adverse events.

**Results:**

Of 65 diabetic foot ulcer wounds from 45 patients assessed for study, 33 wounds from 29 patients were randomized to CAP and 32 wounds from 28 to placebo, with 62 wounds from 43 patients (31 wounds per group) included for final evaluation (mean [SD] age, 68.5 [9.1] years for full sample). Four patients with 5 wounds of 31 (16.1%) wounds in the CAP group and 3 patients with 4 wounds of 31 (13%) wounds in the placebo group were active smokers. CAP therapy yielded a significant increase in wound healing, both in total mean (SD) area reduction (CAP vs placebo relative units, −26.31 [11.72]; *P* = .03) and mean (SD) time to relevant wound area reduction (CAP vs placebo relative units, 10% from baseline, 1.60 [0.58]; *P* = .009). Reduction of infection and microbial load was not significantly different between CAP and placebo. No therapy-related adverse events occurred during therapy; patient’s perceptions during therapy were comparable.

**Conclusions and Relevance:**

In this randomized clinical trial, CAP therapy resulted in beneficial effects in chronic wound treatment in terms of wound surface reduction and time to wound closure independent from background infection.

**Trial Registration:**

ClinicalTrials.gov Identifier: NCT04205942

## Key Points

**Question:**

Does the application of cold atmospheric plasma affect wound healing in patients with diabetic foot ulcers?

**Findings:**

In this randomized clinical trial of 62 diabetic foot ulcers from 43 patients, the application of cold atmospheric plasma significantly improved the healing process in terms of acceleration of wound healing. The healing rate was more pronounced in the plasma group than in the placebo group.

**Meaning:**

In this study, the application of cold atmospheric plasma resulted in improvement in wound healing, which is associated with earlier transition to ambulatory treatment and discharge from the hospital.

## Teaser

This randomized clinical trial assesses the effect of application of cold atmospheric plasma in addition to standard care treatment compared with placebo on wound healing in terms of more rapid and clinical meaningful wound surface regression.

## Introduction

Diabetic foot ulcers (DFUs) are a common complication in patients with diabetes and are associated with increased risk of hospitalization, lower limb amputation, and death and decreases in quality of life.^1,2,3^ The prevalence of DFU in Europe is reported to be 5.1%, with an annual incidence of 2% to 4% in high-income countries.^4,5^ Prevalence is estimated to be between 2% and 10% in Germany.^6^ Rates of DFU are higher in patients with type 2 diabetes (T2D) than those with type 1 diabetes (T1D).^7^ The risk for developing DFU is estimated to be 25%,^8^ with 30% of DFU resulting in lower limb amputation.^9^ Data from Germany in 2014 indicate that 85.6% of patients with minor amputation and 63.7% of patients with major amputation had diabetes.^6^ Data from 2018 on approximately 360 000 patients from general practice in Germany and Austria revealed a prevalence of DFU among patients with T1D of 6.5% and among patients with T2D of 9.9%, with 41.9% (T1D) or 49.4% (T2D) being Wagner grade 2 or 3 and 9.6% (T1D) or 12.5% (T2D) being Wagner grade 4 to 5, indicating the highest grade.^10^ Between 20% and 40% of all chronic DFUs normally heal within 12 weeks, and 50% of the ulcers are healed by 6 months. Approximately 30% of the patients require surgical intervention.^11,12^

The origin of DFU is often a combination of diabetic neuropathy, peripheral arterial disease, foot deformity, and infection.^13^ Ulceration and impaired wound healing are associated with these clinical circumstances.^12^ Early multifactorial assessment and treatment of DFU is necessary for therapeutic success. The earlier that the chronic status of a wound can be resolved, the more efficiently wound therapy can be applied and wound closure can be achieved.

Cold atmospheric plasma (CAP) has been proposed as a tool for various biological and medical applications for its capacity to reduce bacterial load in a wound and to initiate wound healing.^14,15^ In addition to the notable characteristics of CAP, such as charged particles; free electrons and ions; thermal, visible, and UV radiation; and electrical fields, the generation of highly active species at the site of interest may directly function as signaling or redox-reactive molecules.^16^ Biological effects of CAP are largely dependent on plasma-generated reactive species in the gas phase, which diffuse or react with proteins and lipids of cells or tissues. Reactive oxygen species, such as ozone, hydroxyl radicals, superoxide, and singlet oxygen, and reactive nitrogen species, such as nitric oxide or peroxynitrite, are expected to act as active compounds.^17,18^

The objective of this placebo-controlled, patient-blinded study was to assess the effect of application of CAP in addition to standard care treatment compared with placebo on wound healing in terms of more rapid and clinically meaningful wound surface regression. Wound closure progression and microbial analysis were monitored in a time-dependent manner in parallel. Patients’ well-being and subjective perceptions were evaluated during treatment.

## Methods

The Kaltplasma Wund (KPW)-Trial was a prospective, randomized, patient-blinded, placebo-controlled clinical trial in 2 clinics. The study was conducted at the Herz- und Diabeteszentrum Nordrhein Westfalen University Clinic of the Ruhr-University Bochum and the Klinikum Karlsburg. Ethical approval was obtained from the ethics committee of Ruhr-University Bochum, located in Bad Oeynhausen, Germany. The trial was conducted in accordance with the tenets of the Declaration of Helsinki^19^ and Good Clinical Practice guidelines. All participants provided written informed consent prior to participation. This study followed the Consolidated Standards of Reporting Trials (CONSORT) reporting guideline for randomized clinical trials. The trial protocol is available in Supplement 1.

Wounds of the participants were equally randomized to receive either CAP (kINPen Med; neoplas tools GmbH) or placebo intervention by stratified randomization using Research Randomizer software, version 14.0 (Social Psychology Network). Wounds were stratified by patient sex, smoking status (current smoker or nonsmoker), and age (≤68 years and >68 years), resulting in a 2 × 2 × 2 strata. To avoid bias, randomization was undertaken by an independent person. Both treatments were performed at the centers at which patients sought treatment.

Patients with T1D or T2D presenting with at least 1 chronic wound persisting for at least 3 weeks without relevant healing following standard care wound therapy were eligible for this trial. DFU was classified according to the combined Wagner-Armstrong scale^20,21^ by describing the depth of the lesions according to Wagner (0-5, with 0 indicating the shortest depth) with information on infection and peripheral artery disease according to Armstrong (A-D). Thus, superficial wounds and wounds that permeate to tendon presenting with infection but without signs of ischemia (Wagner-Armstrong grade 1B or 2B) were considered eligible for this trial. Infected superficial or infected ulcers extending to tendons or articular capsules were included in this trial. A patient could participate with 1 or more wounds; each wound was randomized separately and seen as a relevant study case. Hospitalized patients between ages 18 and 80 years were included. Exclusion criteria were hemoglobin A_1c_ (HbA_1c_) above 10.0%, concomitant wound treatment with local vacuum therapy or maggot therapy, dialysis, use of topical active antibiotics, concomitant treatment with platelet-rich fibrin, and presence of critical limb ischemia, defined as ankle brachial index below 0.5 or transcutaneous oxygen pressure below 15 mm Hg. Women of childbearing potential without effective contraception, women actively breastfeeding, and active participants in other clinical trials were excluded. Recruitment occurred from August 17, 2016, to April 20, 2019.

Eligible wounds were randomized to receive either placebo or CAP by a plasma argon jet. CAP therapy consisted of active treatment for 5 consecutive days as a once-daily therapy, then every second day for 3 treatments (8 times within 14 days), allowing a 1-day shift in application day. CAP treatment was performed according the manufacturer’s recommendation for 30 s/cm^2^ using sterile spacers to guarantee optimal distance to the wound surface. Patients receiving placebo were treated in a patient-blinded format with the device with the electric field switched off on the device. To maintain procedural blindness for the patient, the sound of the device was added to placebo treatment. Prior to each treatment, microbial samples were taken for analysis of bacterial growth. Questionnaires (EuroQol-5D and the 12-Item Short Form Health Survey) were administered at the beginning and at the end of the treatment period to evaluate a patient’s well-being, as were visual analog scales for pain, well-being, and clinical symptoms. Final evaluation (visit 9) took place 2 to 3 days after the last treatment.

Primary end points of the study were wound surface area reduction, clinical signs and symptoms of infection, and reduction of microbial infection, assessed regularly during treatment and analyzed 2 to 3 days after the last treatment of each patient at visit 9. The same procedure was performed for the secondary end points of time to infection reduction and time to meaningful size reduction, defined as 10% area reduction compared with start of treatment. Quality of life and general well-being were assessed by questionnaires (EuroQol-5D and the 12-Item Short Form Health Survey) during treatment and were defined as secondary end points. In an ad hoc analysis, we analyzed the time to 20% wound area reduction. Adverse events were cumulatively reported.

At each visit, wound size was determined by the physician, and photographs with rulers were taken for blinded evaluation by a third person. Results from the retrospective wound size measurement were cross-checked with the on-visit documentation. The microbial experts were blinded. Data from blinded evaluations were analyzed by a third-party statistician. Questionnaires evaluating quality of life were first scored by a third independent person and then analyzed by the statistician after assigning to the treatment group.

### Statistical Analysis

Significance was set to an α level of .05 and 80% power using a 2-sided unpaired *t* test. For detection of differences between the 2 groups according to wound area reduction after 8 applications with an effect size of 0.75, 29 patient wounds per group needed to be included. Estimating a 10% dropout rate, 66 patient wounds needed to be recruited. The application of a mixed-model approach considering covariables (age, sex, smoking status, wound size, and depth at start of therapy) assumed improvements in statistical power. Sample size calculation was performed using nQuery Advisor + nTerim 2.0 (STATCON GmbH).

Analysis was performed using SAS, version 9.4 (SAS Institute Inc) and Systat, version 13 (Systat Software GmbH). The analysis used the full analysis set containing all intention-to-treat cases with correct inclusion and exclusion criteria. A linear mixed-model was applied for deductive analysis of treatment success and parameters of the primary end point, including patients as a random effect and treatment arm, patient age, sex, and smoking status as constant terms (fixed baseline effects). For analysis of wound reduction, baseline wound size and depth were included as covariables. Log transformation was performed where necessary and indicated as a logistic-linear mixed model. Missing values were added by multiple imputation. For adjustment of multiple comparisons and outcomes, the analysis used a hierarchical test procedure for 3 primary criteria in predefined order (size of wounds, clinical reduction of infection, and proven microbial infection reduction).

For secondary time-to-event end points, the accelerated failure time model was applied. Patients were incorporated as a random parameter, while treatment arm, patient age, sex, and smoking status were considered as fixed elements. Factors were derived from study design (ie, strata of randomization and significant factors from a test of homogeneity). Tests were computed on pre-post data using descriptive and graphical presentations for development between, whereas pre defines the start of treatment and post describes the situation after 8 applications of CAP or placebo treatment.

Symptoms of clinical infection were scored by a system to describe clinical infection at each visit (with 0 indicating no inflammation, 1 indicating a reddened wound, and 2 indicating signs of inflammation), with the highest score being relevant. Microbial load was scored following microbial analysis using 6 levels, with 0 as the lowest and 5 as the highest. Each microbe was scored separately, and a sum score was calculated for total load. Microbial data were log transformed using findings acquired between baseline and visit 9. The analyses of the 12-Item Short Form Health Survey and EuroQol-5D used pre-post score differences as dependent factors and baseline scores as independent factors to compensate for the baseline effect, which was significant in both groups. Descriptive statistics were performed. Data are presented as mean (SD) unless otherwise stated, and *P* values <.05 were regarded as statistically significant.

## Results

**Figure 1.**
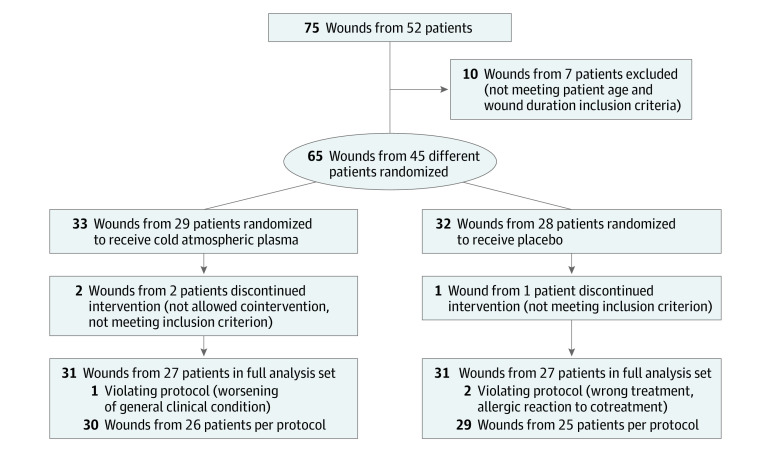
Study Flow Diagram A total of 65 wounds from 45 patients were randomized. A patient could participate with 1 or more wounds in both groups; each wound was randomized separately.

A total of 65 wounds from 45 patients were randomized. A patient could participate with 1 or more wounds in both groups; each wound was randomized separately. After randomization, 33 wounds from 29 patients were assigned to the CAP group and 32 wounds from 28 patients to the placebo group. After predefined exclusion of 3 wounds from 2 patients (HbA1c >10%, concomitant maggot therapy), 62 wounds from 43 patients (CAP, 31 wounds from 27 patients; placebo, 31 wounds from 27 patients [95.4% of included cases]) composed the full analysis set (mean [SD] age, 68.5 [9.1] years). The intervention group included 26 wounds from 23 men (83.9%) and the placebo group included 26 wounds from 23 men (80.6%). Four patients with 5 wounds of 31 wounds (16.1%) in the CAP group and 3 patients with 4 wounds of 31 wounds (12.9%) in the placebo group were from active smokers. Demographic and wound characteristics are presented in Table 1. There were 2 cases in which patients dropped out, were lost to follow up, or withdrew. In 1 case with a silver allergy, the wound was no longer detectable, and 1 treatment could not be performed per protocol because the patient was transferred to the intensive care unit, causing missing values for visits 2 to 9 and for visits 7 to 9 (Figure 1). Therapies were planned at each of the 8 visits after wound inspection. In the CAP group, 100% of planned standard care therapy and 99.1% of planned study treatment were applied; in the placebo group, 99.6% of standard care therapy and 96.4% of study treatment were applied.

**Table 1.** Demographic Data and Wound Status at Baseline From the Analysis Cohort

Parameter	No. (%)	
	CAP	Placebo
No. of wounds randomized^a^	33	32
No. of wounds analyzed^b^	31	31
No. of wounds in men	26 (83.9)	25 (80.6)
No. of wounds in women	5 (16.1)	6 (19.4)
Patient age, mean (SD), y	68.3 (9.5)	68.7 (8.8)
No. of wounds in age group ≤68 y	15 (48.4)	14 (45.2)
No. of wounds in age group >68 y	16 (51.6)	17 (54.8)
No. of wounds from current smokers	5 (16.1)	4 (12.9)
Wound status		
Wound duration, median (95% CI), d	90 (85.1-701.3)	60 (64.9-389.1)
Wagner-Armstrong classification^c^		
1B (superficial infected wound)	25 (80.6)	24 (77.4)
2B (infected wound permeating to tendon)	6 (19.4)	7 (22.6)
Wound surface, median (95% CI), cm^2^	2.82 (1.06-5.89)	1.32 (0.50-2.45)
Wound base (multiple selections possible)		
Fibrinous	10 (32.3)	5 (16.1)
Granulating	15 (48.4)	13 (41.9)
Bradytrophic	11 (35.5)	14 (45.2)
Wound edge (multiple selections possible)		
Reddened	9 (29.0)	6 (19.4)
Swollen	11 (35.5)	7 (22.6)
Inflamed	10 (32.3)	11 (35.5)
Hyperkeratotic	7 (22.6)	7 (22.6)
Other	5 (16.1)	7 (22.6)
Wound environment (multiple selections possible)		
Reddened	8 (25.8)	6 (19.4)
Swollen	2 (6.5)	1 (3.2)
Inflamed	4 (12.9)	6 (19.4)
Nonirritant	14 (45.2)	13 (41.9)
Other	4 (16.1)	7 (22.6)
Presence of wound pain		
Yes	13 (41.9)	9 (29.0)
No	18 (58.1)	21 (67.7)
Not specified	0	1 (3.2)
Wound colonization		
*Corynebacterium* species	13 (17.57)	9 (16.36)
*Proteus* species	3 (4.05)	4 (7.27)
* Pseudomonas aeruginosa*	3 (4.05)	3 (5.45)
* Staphylococcus aureus*	13 (17.57)	7 (12.73)
Other staphylococci	22 (29.73)	21 (38.18)
*Streptococcus* species	5 (6.76)	1 (1.82)
Other	15 (20.27)	10 (18.18)

^a^ Number of patients was 29 for CAP and 28 for placebo.

^b^ Number of patients was 27 for CAP and 27 for placebo.

^c^ The combined Wagner-Armstrong scale describes the depth of the lesions according to Wagner (0-5, with 0 indicating the shortest depth) with information on infection and peripheral artery disease according to Armstrong (A-D).

### Primary End Points

**Figure 2.**
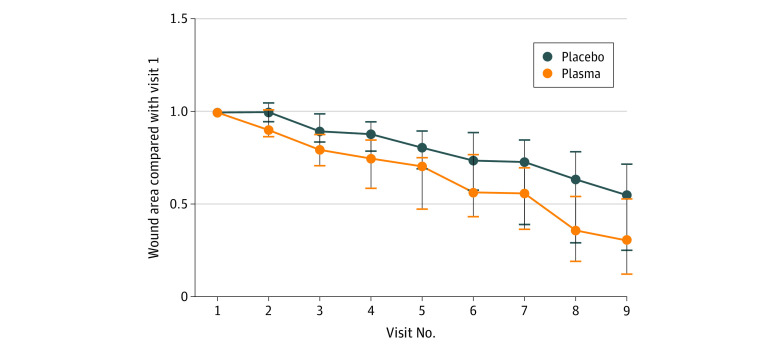
Primary End Point of Wound Size Reduction, Given as Reduction in Relation to Start of Therapy Depicted are medians and 95% CIs. Per end point analysis, there was significant difference in wound size at visit 9.

Both treatments resulted in wound surface reduction. The CAP group presented with significant improvement compared with placebo therapy after visit 9 (CAP: remaining wound area: 30.5%; 95% CI, 12.3%-53.5% vs placebo: remaining wound area: 55.2%; 95% CI, 25.2%-72.0%; estimated mean [SD] value, CAP vs placebo: −26.31 [11.72]; *P* = .03) (Figure 2 and Table 2).

**Table 2.** Primary Study End Points

Parameter	CAP		Placebo		*P* value
	Start of therapy	End of therapy	Start of therapy	End of therapy	
Wound area, median (95% CI), %	100	30.5 (12.3-53.5)	100	55.2 (25.2-72.0)	.03
Clinical infection mean score, No.^a^					.91
0	5	26	4	25	NA
1	16	3	20	4	
2	10	1	7	1	
Mean (SD)	1.16 (0.54)	0.17 (0.29)	1.10 (0.41)	0.13 (0.23)	
Quantitative microbial infection score, mean (SD)^b^	4.10 (4.57)	1.94 (2.22)	3.16 (4.66)	1.58 (2.25)	.59

Abbreviation: NA, not applicable.

^a^ For clinical infection: score 0, nonirritant; score 1, reddened; and score 2, inflamed.

^b^ For microbial analysis of colonization; a score of 0, no growth; 1, detection after enrichment; 2, little growth; 3, intermediate growth; 4, plentiful growth; and 5, massive growth was applied. The sum of the score of all germs detected was calculated as the infection score.

Scores representing clinical infection decreased equally in both groups, yielding comparable results at the end of treatment. Use of the logistic-linear mixed-model detected no significant difference after baseline correction; reduction of clinical infection was comparable in both groups (CAP, 85% vs placebo, 88%; *P* = .91).

Microbial load was analyzed as described in the Methods section. This analysis underlined the observed clinical effect and generated a similar curve. Both CAP and placebo treatments reduced microbial load. Wounds in the CAP group started with higher bacterial load score (mean, 4.19) than did placebo wounds (mean, 3.56), yielding scores of 1.94 and 1.58 after treatment, respectively. There was no significant difference in reduction of microbial load between groups (CAP, 53% vs placebo, 50%; *P* = .59) (Table 2). When the analysis was limited to wounds with an area of at least 1 cm^2^ to avoid unintentional dermal probing in ad hoc analysis, the difference in microbe reduction was not significant (CAP, −1.16 vs placebo, −0.42; *P* = .18).

### Explorative Secondary End Points

**Figure 3.**
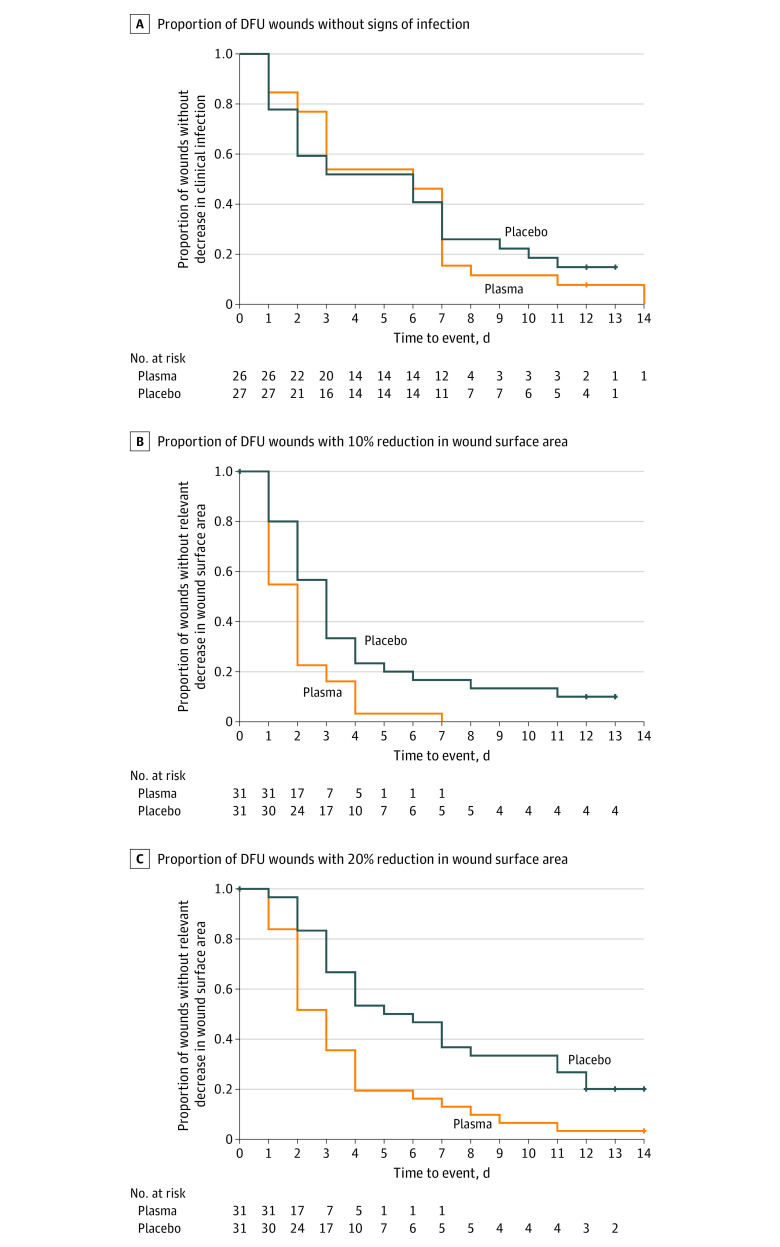
Key Secondary End Points A, Proportion of wounds without signs of clinical infection during treatment. B, Proportion of wounds with 10% reduction in wound surface area during treatment. C, Proportion of wounds with 20% reduction in wound surface area during treatment (ad hoc analysis). DFU indicates diabetic foot ulcer.

The time to infection reduction was comparable between both treatment arms. Analysis followed the accelerated failure time model considering patient as a random component and treatment and baseline strata as fixed components (Figure 3A).

A meaningful reduction in wound surface was defined as 10% before analysis. All wounds in the CAP group reached this end point, whereas 3 wounds in the placebo group did not. As this predefined criterion produced no variation in the CAP group, we performed an ad hoc analysis with the meaningful wound reduction criterion set to 20%.

Dynamics of ulcer healing were analyzed by comparing the percentage reduction in wound size between the groups (Figure 3A). Time to reach meaningful reduction was plotted in Kaplan-Meier curves, and progress was analyzed via accelerated failure time model analysis. Within the CAP group, a significant earlier mean (SD) wound reduction was detected when applying the 10% criterion (−1.60 [0.58]; *P* = .01) and the 20% criterion (−1.83 [0.53]; *P* = .001) (Figure 3B and C).

With regard to quality of life, the questionnaires EuroQol-5D and 12-Item Short Form Health Survey yielded comparable mean (SD) score shifts in life quality in both groups (CAP vs placebo: −0.07 [09]; *P* = .42 and 1.25 [0.90]; *P* = .16, respectively). Analysis by visual analog scales (scored from 0-10, with 0 indicating no effects and 10 indicating most effects) revealed that effects on symptoms like sensation of prickling, heat, coldness, numbness, furry feeling, smell, and exudation were not significantly different between groups. Comparable results were achieved for pain sensation and general well-being.

Expected adverse events during treatment included scar formation, skin irritation, bleeding, and proliferation. In both groups, such events were equally distributed (7 for CAP and 6 for placebo). In total, 105 unexpected adverse events (58 for CAP and 47 for placebo) were documented during the active trial phase (eTable in Supplement 2). These events are equally distributed in both treatment arms if qualified by following the Medical Dictionary for Regulatory Activities System Organ Class. Nine adverse events (CAP, 4; placebo, 5) have been associated with therapy; 21 adverse events have possible association with therapy (CAP, 13; placebo, 8). Two severe unexpected adverse events occurred during the study: 1 patient experienced aortic valve stenosis (1 wound per group) and 1 patient experienced cardiopulmonary resuscitation (CAP group). All severe unexpected adverse events were qualified as not being related to study therapy or procedures and resolved shortly after occurrence.

## Discussion

To our knowledge, this is the first randomized, prospective, placebo-controlled clinical trial analyzing the effect of CAP therapy on wound healing in DFU in a population-based, representative cohort of patients regarding reduction of wound size. From experimental biology, the antimicrobial effects of CAP have been reported after application of cold plasma, present both in the biological environment and on artificial surfaces.^22,23^ From first clinical applications, this effect was considered as a key factor in the application in wounds presenting with bacterial load. These effects are apparent directly after treatment.^14^ However, CAP therapy revealed beneficial effects in chronic wound treatment in terms of wound surface reduction, granulation, and time to wound closure and proved to be more relevant for wound healing than its antimicrobial effects. A 2008 analysis^24^ in wound models reported improvement of microcirculation and granulation after CAP treatment. Predictive models for achieving/prognosing wound healing were discussed when considering healing after 12 weeks of treatment. Cardinal et al^24^ proposed algorithms to calculate wound healing time and showed a clear difference between nonhealing and healing wounds at the level of 10% wound area reduction. We were more focused on the short time effect at therapy initiation and therefore on early wound area reduction and took the 10% reduction mark as clinically meaningful. An ad hoc analysis with a cutoff point set to 20% reduction was performed to substantiate effect data. Wound surface area and depth were included as covariables into the statistical analysis to eliminate size-related and depth-related effects to overcome effects of different wound size.

Our results indicate that CAP therapy exerts beneficial effects on wound healing independently from bacterial load reduction, which was first assumed from in vitro experiments analyzing freshly treated, artificially infected wounds and technical surfaces.^25,26,27^ CAP-treated wounds significantly evolved into a healing process as they reached the 10% and the 20% surface reduction mark earlier, irrespective of bacterial load reduction and infection status, as these 2 factors were not significantly different in both treatments. With this result, the trial reached its primary end point. These results support the hypothesis that CAP effects do not primarily rely on antimicrobial effects but directly activate quiescent chronic wounds.

Both CAP and placebo treatments reduced microbial load. Because of high heterogeneity and low abundance in single cases, this reduction did not reach statistical significance and cannot be considered as a factor in clinical outcome. All wounds received standard wound care procedures including systemic antibiotic treatment if indicated, regular wound debridement, local disinfection, off-loading, and moist wound care. These procedures may account for the microbial reduction seen in both groups and may dilute the CAP effect on microbial load. Results regarding reduction in microbial load focus on acute effects directly after application of CAP,^27,28^ whereas in our study, microbial load was measured before each treatment and not directly afterward. This process may account for differences in antimicrobial final treatments. Colonization with dermal microbiota is common in DFU as wound dressings may dislocate and could contribute to mixing dermal with wound microbiota.

CAP therapy was well tolerated among treated patients, and few treatment-related adverse or serious adverse events were reported. Patients are still in follow-up for at least 5 years (resulting in an end to the study in April 2024) to generate long-term observational data on the durability of the effect and safety aspects.

Results of this trial are of clinical relevance for both patients and health care professionals. Turning a chronic wound into a healing wound with the application of CAP may be associated with the duration of hospitalization. CAP treatment application does not require specialized centers. The jet technique used in this study may adapt to heterogenous wound depths in chronic wounds. The intervention device, a contact-free and environment-independent application method, used argon gas instead of ambient air for production of a more stable plasma, and did not use the wound surface as a counter electrode.

### Limitations

This study has limitations. Although the number of patients was small, producing wide confidence intervals, the study was statistically powered to resolve the key question. The study did not show effects on microbial reduction as expected, which may result from procedural issues which included systemic antibiotic therapy as standard care therapy and the determination of bacterial load at each dressing change on every second day. Recolonization must be considered as a limitation and should be considered in further trials analyzing antimicrobial effects. Randomization was performed on the wound level, and different wounds were assigned to both treatment groups randomly. The analysis operated on a wound level and incorporated the patients as random effect in mixed models.

## Conclusions

To our knowledge, this is the first placebo-controlled blinded prospective trial applying CAP in DFU-related chronic wounds, suggesting that accelerated wound healing resulted in significant wound size reduction. No therapy-related serious adverse events occurred. Application of CAP could potentially result in earlier discharge from the hospital, which may be relevant for patients and health care professionals.

## Notes

Supplement 1. **Trial Protocol**

Supplement 2. **eTable.** Classification of AEs According to MedDRA-System Organ Code (SOC)

Supplement 3. **Data Sharing Statement**
