# Numeric Errors in Figure 3

**DOI:** 10.1001/jamanetworkopen.2020.24427

**Published:** 2020-10-02

In the Original Investigation titled “Effect of Cold Atmospheric Plasma Therapy vs Standard Therapy Placebo on Wound Healing in Patients With Diabetic Foot Ulcers: A Randomized Clinical Trial,”^1^ published on July 16, 2020, there were numeric errors in Figure 3. Numbers of patients at risk that should have appeared in panel B appeared in panel C, and numbers that should have been listed in panel C were not included. Graphs presented are not affected by correction; data analysis and conclusions drawn are unchanged. This article has been corrected.^1^
