# Supplementary material for: Effect of Cold Atmospheric Plasma Therapy vs Standard Therapy Placebo on Wound Healing in Patients With Diabetic Foot Ulcers: A Randomized Clinical Trial
Source: JAMA Netw Open. 2020 Jul 16;3(7):e2010411. doi: 10.1001/jamanetworkopen.2020.10411 (PMC7366186; doi:10.1001/jamanetworkopen.2020.10411)
Supplement: Supplement 1. — Trial Protocol [file jamanetwopen-3-e2010411-s001.pdf]

Study Protocol

COLD PLASMA THERAPY FOR ACCELERATION OF WOUND HEALING IN DIABETIC FOOT

KPWTRIAL

Principal investigator

Univ. Prof. Dr. Dr. D. Tschöpe
Herz- und Diabeteszentrum NRW
Diabeteszentrum
Georgstr. 11
32545 Bad Oeynhausen
☏ +49-(0)5731 – 972292
Fax: +49-(0)5731 – 971967
Email: diethelm.tschoepe@ruhr-uni-bochum.de

Investigator

Oberärztin Dr. medic Tania-Cristina Costea
Herz- und Diabeteszentrum NRW
Diabeteszentrum
Georgstr. 11
32545 Bad Oeynhausen
☏ +49-(0)5731 – 973710
Fax: +49-(0)5731 – 972122
Email: tcostea@hdz-nrw.de

Study coordinator

Dr. rer. nat. B. Stratmann
Herz- und Diabeteszentrum NRW
Diabeteszentrum - Forschung
Georgstr. 11
32545 Bad Oeynhausen
☏ +49-(0)5731 – 973768
Fax : +49-(0)5731 – 972410
Email: bstratmann@hdz-nrw.de

Add. Study site :
Klinikum Karlsburg, Greifswalder Str. 11, 17495 Karlsburg
Prüfer : Prof. Dr. Wolfgang Kerner

Study-No.: KPW2016-1.1

Content

SYNOPSIS 3

1. INTRODUCTION 5

1.1 Therapeutic interventions 5

2. OBJECTIVES OF THE STUDY 6

2.1 primary objectives 7

3 STUDYDESIGN 7

3.1 General design 7

3.2 Sample Size 7

3.3 Time schedule 7

4 PATIENT SELECTION 8

4.1 inclusion criteria 8

4.2 exclusion criteria 8

4.3 Documentation of patient data 8

5 STUDY MATERIALS 8

6 CONCOMITANT THERAPY, THERAPY BEYOND END OF STUDY 8

7 POTENTIAL RISK 9

8 PREMATURE DISCONTINUATION 9

8.1 for patients 9

8.2 for the trial 9

9 REFERENCES 10

10 APPENDIX 11

10.1 CE-Certificate Plasma Jet 11

Synopsis

COLD PLASMA THERAPY FOR ACCELERATION OF WOUND HEALING IN DIABETIC FOOT

V1.3 as of 05/JUL/2016	STUDY-NO.: KPW2016-1.1
Objective	People with diabetes mellitus present with a higher risk of developing chronic wounds. A reduced pain sensation resulting from neuropathy often accounts for wounding. Therefore, people with diabetes show higher risks for late complications like limb amputation, which causes loss of life quality and increased mortality. Main objective of this study is to determine, whether application of cold plasma could significantly improve wound heal-ing.
Study design	This study will be conducted as a bi-center, randomized, patient-blinded study with a certified medical device. For determination whether cold plasma application in patients with diabetes and diabetic foot Wagner-Armstrong stage 1B or 2B as well as ulcus curis may accelerate wound healing, people presenting with either type 1 or type 2 diabetes mellitus will be randomized to plasma or placebo therapy, respectively. Each group should be comparable according to age, gender and ulcus-stadium.
No. of patients/ duration of study	A dropout rate of 10% is assumed for this study. 66 patient wounds will be recruited to result in 60 evaluable data sets. Study procedures will be per-formed at HDZ NRW in Bad Oeynhausen and at Klinikum Karlsburg in Karlsburg. Patient wounds will be equally assigned to both treatment groups (plasma and placebo) via stratified randomization. Patient being hospitalized for treatment of diabetic foot (Wagner-Armstrong stage 1B or 2B) are in focus of the study which is carried out in a hospital setting. The study is planned to take approximately 8 months comprising 8 applications (5 on consecutive days, 3 afterwards in every second day manner). Follow up evaluations are planned after 1, 2 and 5 years after the first application to evaluate therapeutic side effects and long-term effects of the therapy.
Key inclusion criteria	▪ Diabetes mellitus Typ 1/ Diabetes mellitus Typ 2<br>▪ HbA1c ≤ 10%<br>▪ Age 18 – 80 years<br>▪ Ulcus Wagner-Armstrong 1B bzw. 2B (persistent for at least 3 weeks, no healing tendency)
Key exclusion criteria	▪ Participation in another clinical study with an IP during the last month prior to enrolment<br>▪ Wound treatment by local VAC-therapy, maggot therapy<br>▪ Essential dialysis therapy<br>▪ Application of topical antibiotics<br>▪ Treatment with platelet rich fibrin (PRF)<br>▪ Critical limb ischemia, defined as ankle brachial index below 0.5 or transcutaneous oxygen pressure below 15 mmHg.

Time schedule

	Day 1	Day 2-4	Day 5	Day 7	Day 9	Day 12	Day 14	Year 1	Year 3	Year 5
Visit-No.	1	2-4	5	6	7	8	9	10	11	12
In-/Exclusion criteria	x	-	-	-	-	-	-	-	-	-
Informed consent	x	-	-	-	-	-	-	-	-	-
Randomization	x	-	-	-	-	-	-	-	-	-
Wound documentation*	x	x	x	x	x	x	x	x	x	x
Mikrobiology (swap)	x	-	x	x	-	x	x	-	-	-
Local infection signs	x	x	x	x	x	x	x	-	-	-
Sampling biomaterial wound dressings	x	x	x	x	x	x	x	-	-	-
Questionnaire SF-12	x	-	-	-	-	-	x	x	x	x
Questionnaire EQ5D	x	-	-	-	-	-	x	x	x	x
Visual analog scales	x	-	-	x	-	-	x	x	x	x
treatment	x	x	x	x	x	x	-	-	-	-
Documentation of side effects	x	x	x	x	x	x	x	x	x	x
Standard wound care	x	x	x	x	x	x	x	-	-	-

* before therapy, surface measurement and photo documentation; at V10, V11, V12 documentation at original site were wound has been, if absent

1. Introduction

People with diabetes mellitus present with increased risk for superficial chronic wounds due to the presence of nerve impairments and reduced pain sensation (1, 2). 70-100% of patients with chronic wounds show symptoms of peripheral neuropathy. 0.8 to 10% of people with diabetes suffer from diabetic foot (DF), the yearly occurrence rate is 2,2–5,9% (3). Up to 30% of all diabetics show sensomotoric or autonomic neuropathy. All functional aspects of peripheral nerves are damaged by prolonged hyperglycemia. Because sensation for contact, pain and temperature is reduced or even lost, such stressors are not recognized, own protection mechanisms are lost. In addition, deep sensibility is reduced as well. Disequilibrium and imbalanced walking with abnormal biomechanical load induces pressure ulcerations (4, 5).

Co-existence of peripheral artery disease (PAD) and polyneuropathy causes less perfusion and nutrition in the wound area resulting in reduced healing. In most cases neuropathy and PAD, together, account for wound chronification. Superficial microbial load is common in diabetic foot, requiring antiseptic treatment and regular changes of dressings. Due to decreased pain sensation caused by neuropathy PAD is often diagnosed at advanced stages with concomitant occurrence of ulcerations or gangrene. Sometimes even simple wounds or short trauma develop to chronic wounds (6).

1.1 Therapeutic interventions

Off-loading

Beside improvements in the perfusion off-loading represents a key element in modern wound treatment. Bed-rest, use of crutches, or wheel chair are commonly applied. Orthopedic shoes up to total contact casts (TCC) are prescribed; orthoses are of comparable function if consequently worn by the patient (3).

Wound treatment

Debridement of avital necrotic wound tissue is essential in modern wound treatment to prepare optimal wound conditions. Debridement can be performed manually, enzymatically, or biologically using maggot therapy. The extent of procedures varies from case to case and may comprise local removal of affected fiber or bone. Local vacuum therapy can be applied to stimulate wound granulation and to remove exudation (7).

Wound desinfection

Each change of dressings should include antiseptic wound treatment (if infection is present) at least wound cleaning for biofilm removal. Moisture wound dressings are common for chronic wound treatment, a local temperature of at least 28°C is necessary for cell proliferation. Systemic antibiotic treatment has to be applied if clearly detectable proliferative bacterial load is present, whereas topic antibiotics are less effective (8).

Cold atmospheric plasma

Physical plasma is a highly reactive, ionized gas. Combination of several effective components of the plasma, such as reactive species derived from oxygen and nitrogen, a mild UV radiation, a mild current flow, and an increased temperature at the application site account for the antiseptic effect (8, 9). Through the interaction with molecules from air, body fluids, or target tissue active components are distributed in the target zone (7, 10). Currently, the exact mechanisms of action are poorly understood, but reactive oxygen species are thought to play a major role (11). Physical deteriorations caused by UV radiation or thermal stress are neglectable (7, 12).

Application of cold atmospheric plasma via plasma jet (kINPen® MED) is suitable for chronic, infected wounds like ulcus cruris or decubital ulcus, and pathogenic skin irritations (Acne vulgaris or Tinea pedis).

During application wound healing is supported by improved cell proliferation and antimicrobial effects. The plasma jet produces a handwarm plasma of good compatibleness, that is painfree and easy to handle (7). During clinical routine use no negative side effects have been detected.

2. Objectives of the study

The objective of the study is to demonstrate an improvement in wound healing in diabetic foot by application of cold atmospheric plasma. Patient with either type 1 or type 2 diabetes mellitus will be randomized to receive plasma or placebo therapy. Each wound will be randomized separately. Eligible participants present with at least one chronic wound persisting for at least three weeks without healing tendency under standard wound care.

Study flow scheme

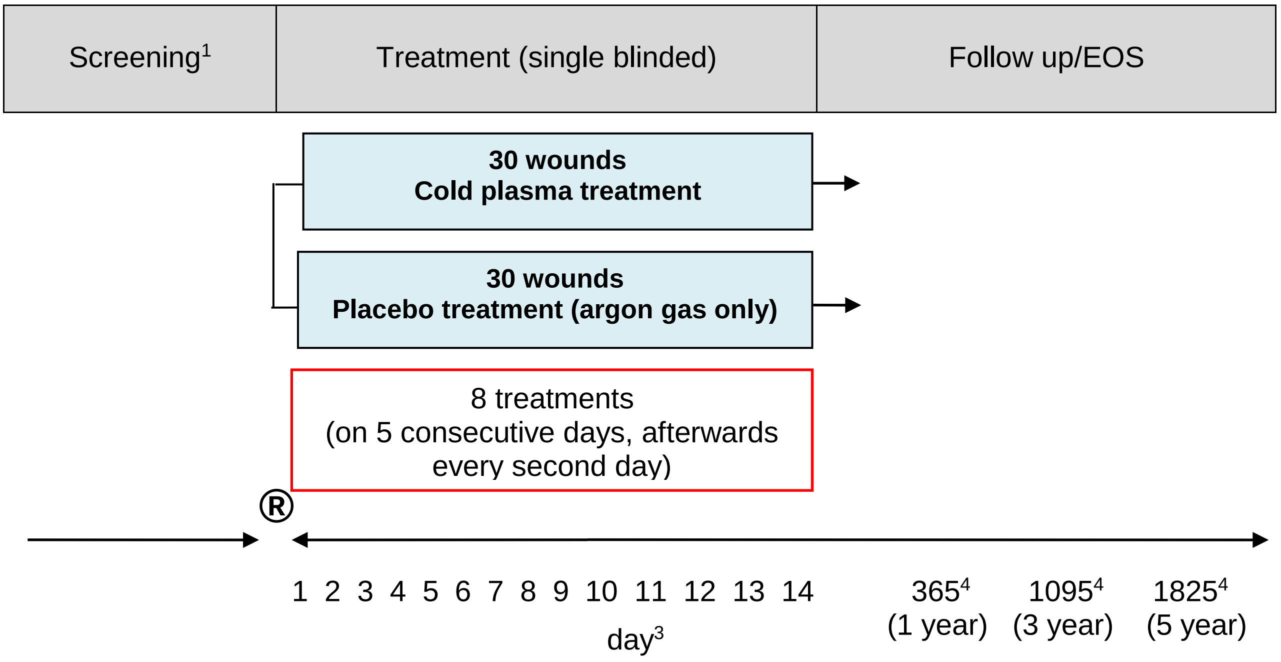

R=Randomization
EOS= End of Study

[1] Screening: Wound status, sample of materials
[2] EOS: questionnaires, Wound status, sample of materials
[3] time frame ±1 day
[4] time frame ± 30 days

2.1 primary objectives

- change in wound surface area
- change in signs of clinical infection
- change in microbial load

2.2 Secondary Outcome Measures

- time to significant wound surface area change (time to 10% reduction of wound surface compared to treatment start)
- total wound surface change during treatment
- time to change in wound infection
- changes in Quality of life (EQ5D questionnaire)
- changes in Quality of life (SF12 questionnaire)
- Evaluation of syptoms during therapy (visual analog scale)
- treatment related side effects - formation of keloids, presence of skin irritation, local bleeding, proliferative skin reaction

3 Study-design

3.1 General design

This study is a prospective, randomized, patient-blinded, placebo controlled bi-center study. The study will evaluate whether application of cold atmospheric plasma will accelerate wound healing in diabetic foot patients presenting with wound Wagner-Armstrong 1B or 2B.

3.2 Sample Size

Hospitalized patients fulfilling eligibility criteria at HDZ NRW or Klinikum Kalrlsburg are recruited for the trial. After informed consenting wounds will be randomized to one or the treatment arm applying stratified randomization, considering patient's age, gender and smoking status. 30 wounds per group shall be analyzed. Considering a dropout rate of 10% 66 wounds in total have to be recruited.

3.3 Projected time schedule

Preparation and recruitment:	continuously
Data analysis:	3 Months
Total trial duration:	8 Months, incl. Follow up: 5 years, 8 Months
Start of study:	July 2016
End of study incl. follow up:	December 2021

4 Patient selection

4.1 inclusion criteria

• Type 1 or Type 2 Diabetes mellitus
• HbA1c ≤ 10%
• at least one chronic wound persisting for at least three weeks without healing tendency following standard care wound therapy (Armstrong-Wagner Grade 1B or 2B)

4.2 exclusion criteria

• concomitant wound treatment with local vacuum therapy or maggot therapy
• dialysis
• use of topical active antibiotics,
• concomitant treatment with platelet rich fibrin (PRF),
• presence of critical limb ischemia defined as ankle brachial index below 0.5 or transcutaneous oxygen pressure below 15 mmHg.
• participation in another clinical trial
• women of child bearing potential without effective contraception or active breastfeeding

4.3 Documentation of patient data

Before randomization eligibility criteria will be checked by the investigator. Further data documentation comprises:

- Patient's initials
- Date of birth
- gender
- background medication
- medical history
- concomitant diseases

5 study materials

Patient's well-being will be evaluated at the beginning, during and at the end of the study using questionnaires (EQ5D, SF12, Visual analog scales to evaluate clinical symptoms, pain, and well-being).
At HDZ NRW wound dressings will be sampled at each visit to analyze wound healing on the level of protease, growth factors and markers of inflammation.

6 concomitant therapy, therapy beyond end of study

The concomitant therapy will be recorded and updated regularly at each visit using study specific forms. Wound therapy will be monitored either. At distinguished visits wound swaps will be taken and analyzed for microbial load. At each visit the investigator will be asked to judge clinical infection of the wound upon signs and symptoms.

At the end of the treatment phase the investigator will develop an individual plan for further treatment of the patient, either in an ambulatory or clinical setting.

7 potential risk

According to current knowledge application of cold atmospheric plasma is not associated with negative clinical side effects. Thus, no additional risk for the participants is to be awaited. Cold plasma therapy is used for the treatment of superficial skin diseases like acne, scars or cicatrices. Even from the therapy of tumors positive effects have been described. Cellular impairments caused by UV-radiation or thermal stress was excluded in clinical studies (7, 12). The application of cold plasma will be performed only by trained personal at both study sites. The device in use is CE-certified and licensed for the superficial skin treatment (kINPen® Med, Appendix 1).

Treatment is planned as an ad on treatment to standard wound care performed during the hospital stay of the patients. Thus, for both groups, plasma as well as placebo, no further risk is awaited. To current knowledge, patients feel no pain during treatment and potential therapy associated risks are of low occurrence.

8 premature discontinuation

8.1 for patients

Patients' participation in this trial is voluntarily. All patients may at any point in the trial withdraw from the trial, without giving any reasons.

Possible reasons could be:

- withdraw of consent
- exclusion by investigator due to medical reasons
- pregnancy
- inadequate compliance of the patient (e.g. Off-loading of the wound)
- violation of in- or exclusion criteria
- worsening of wound condition, affording additional treatment
- newly detected disease, affecting the study intended treatment

8.2 for the trial

The trial may be interrupted or stopped by the principal investigator taking into account the benefit/risk-ratio.

Possible reasons are:

- New knowledge on possible risks or facts, that make a re-evaluation of benefit/risk ratio necessary, ending up in a negative result
- Occurrence of severe unexpected treatment related adverse events

9 References

1. Salvotelli L, Stoico V, Perrone F, Cacciatori V, Negri C, Brangani C, et al. Prevalence of neuropathy in type 2 diabetic patients and its association with other diabetes complications: The Verona Diabetic Foot Screening Program. J Diabetes Complicat. 2015;29(8):1066-70.
2. Kim PJ, Steinberg JS. Complications of the Diabetic Foot. Endocrin Metab Clin. 2013;42(4):833-47.
3. Morbach S, Müller, E. , Reike, H., Risse, A., Rümenapf, G., Spraul, M. . Diabetisches Fußsyndrom. Diabetologie und Stoffwechsel 2014;9(S02):169-77.
4. Gupta A, Gupta Y. Diabetic Neuropathy: Part 1. J Pak Med Assoc. 2014;64(6):714-8.
5. Pasnoor M, Dimachkie MM, Kluding P, Barohn RJ. Diabetic Neuropathy Part 1 Overview and Symmetric Phenotypes. Neurol Clin. 2013;31(2):425-+.
6. Forsythe RO, Hinchliffe RJ. Management of peripheral arterial disease and the diabetic foot. J Cardiovasc Surg. 2014;55(2):195-206.
7. Kong MG, Kroesen G, Morfill G, Nosenko T, Shimizu T, van Dijk J, et al. Plasma medicine: an introductory review. New J Phys. 2009;11.
8. Kieft IE, Broers JLV, Caubet-Hilloutou V, Slaaf DW, Ramaekers FCS, Stoffels E. Electric discharge plasmas influence attachment of cultured CHO k1 cells. Bioelectromagnetics. 2004;25(5):362-8.
9. Heinlin J, Isbary G, Stolz W, Morfill G, Landthaler M, Shimizu T, et al. Plasma applications in medicine with a special focus on dermatology. J Eur Acad Dermatol. 2011;25(1):1-11.
10. Kalghatgi S, Kelly CM, Cerchar E, Torabi B, Alekseev O, Fridman A, et al. Effects of Non-Thermal Plasma on Mammalian Cells. Plos One. 2011;6(1).
11. Ahn HJ, Kim KI, Kim G, Moon E, Yang SS, Lee JS. Atmospheric-Pressure Plasma Jet Induces Apoptosis Involving Mitochondria via Generation of Free Radicals. Plos One. 2011;6(11).
12. Partecke LI, Evert K, Haugk J, Doering F, Normann L, Diedrich S, et al. Tissue Tolerable Plasma (TTP) induces apoptosis in pancreatic cancer cells in vitro and in vivo. Bmc Cancer. 2012;12.

This certificate is valid until: 26 February 2018
Hamburg, 03 May 2013

Process No.: QS - 7197
Certificate No.: 7197GB414130503
MEDCERT Identification No.: 0482

MEDCERT Certification Body
(Markus Bianchi)

Akkreditiert durch
Zentralstelle der Länder
für Gesundheitsschutz
bei Arzneimitteln
und Medizinprodukten
ZLG-ZQ-983.94.13

10.1 CE-Certificate Plasma Jet

EC Certificate of Conformity

The Notified Body

MEDCERT Zertifizierungs- und Prüfungsgesellschaft für die Medizin GmbH
Pilatuspool 2 – 20355 Hamburg – Germany

herewith confirms that the company

neoplas tools GmbH
Walter-Rathenau-Str. 49a – 17489 Greifswald
Germany

has introduced and applies a Quality Management System
for the products / product categories

Plasma devices for wound cleansing
and therapy of skin diseases

An Audit demonstrated compliance of the Quality Management System with

Annex V

of the **Council Directive 93/42/EEC concerning Medical Devices**.

The certification assumes that the company is applying and maintaining its Quality Management System according to the above given annex. The license of certification is subject to surveillance by MEDCERT.

This certificate is valid until: 26 February 2018
Hamburg, 03 May 2013

Process No.: QS - 7197
Certificate No.: 7197GB414130503
MEDCERT Identification No.: 0482

MEDCERT Certification Body
(Markus Bianchi)

Akkreditiert durch
Zentralstelle der Länder für Gesundheitsschutz bei Arzneimitteln und Medizinprodukten
ZLG-ZQ-983.94.13
