# Supplementary material for: Effect of Cold Atmospheric Plasma Therapy vs Standard Therapy Placebo on Wound Healing in Patients With Diabetic Foot Ulcers: A Randomized Clinical Trial
Source: JAMA Netw Open. 2020 Jul 16;3(7):e2010411. doi: 10.1001/jamanetworkopen.2020.10411 (PMC7366186; doi:10.1001/jamanetworkopen.2020.10411)
Supplement: Supplement 2. — eTable. Classification of AEs According to MedDRA-System Organ Code (SOC) [file jamanetwopen-3-e2010411-s002.pdf]

# Supplementary Online Content

Stratmann B, Costea T-C, Nolte C, et al. Effect of cold atmospheric plasma therapy vs standard therapy placebo on wound healing in patients with diabetic foot ulcers: a randomized clinical trial. *JAMA Netw Open*. 2020;3(7):e2010411. doi:10.1001/jamanetworkopen.2020.10411

**eTable.** Classification of AEs according to MedDRA-System Organ Code (SOC)

This supplementary material has been provided by the authors to give readers additional information about their work.

© 2020 Stratmann B et al. *JAMA Network Open.*

eTable. Classification of AEs according to MedDRA-System Organ Code (SOC)

| | Code | Cold Plasma | | Placebo | | Total | |
|---|---|---|---|---|---|---|---|
| | 100… | N | % | N | % | N | % |
| Gastrointestinal disorders | 17947 | 5 | 8.62 | 0 | 0.0 | 5 | 4.76 |
| General disorders and administration site conditions | 18065 | 26 | 44.83 | 21 | 44.86 | 47 | 44.76 |
| Injury, poisoning and procedural complications | 22117 | 12 | 20.69 | 10 | 21.28 | 22 | 21.20 |
| Metabolism and nutrition disorders | 27433 | 0 | 0.0 | 3 | 6.38 | 3 | 2.86 |
| Musculoskeletal and connective tissue disorders | 28395 | 3 | 5.17 | 2 | 4.26 | 5 | 4.76 |
| Nervous system disorders | 29205 | 0 | 0.0 | 1 | 2.13 | 1 | 0.95 |
| Respiratory, thoracic and mediastinal disorders | 38738 | 3 | 5.17 | 2 | 4.26 | 5 | 4.76 |
| Skin and subcutaneous tissue disorders | 40785 | 8 | 13.79 | 4 | 8.51 | 12 | 11.43 |
| Surgical and medical procedures | 42613 | 1 | 1.72 | 2 | 4.26 | 3 | 2.86 |
| Vascular disorders | 47065 | 0 | 0.0 | 2 | 2.13 | 1 | 0.95 |
| Peripheral artery occlusion | 57525 | 0 | 0.0 | 1 | 2.13 | 1 | 0.95 |
| | | 58 | 100 | 47 | 100.0 | 105 | 100.0 |

© 2020 Stratmann B et al. *JAMA Network Open.*
