# Supplementary material for: Effect of Cold Atmospheric Plasma Therapy vs Standard Therapy Placebo on Wound Healing in Patients With Diabetic Foot Ulcers: A Randomized Clinical Trial
Source: JAMA Netw Open. 2020 Jul 16;3(7):e2010411. doi: 10.1001/jamanetworkopen.2020.10411 (PMC7366186; doi:10.1001/jamanetworkopen.2020.10411)
Supplement: Supplement 3. — Data Sharing Statement [file jamanetwopen-3-e2010411-s003.pdf]

# Data Sharing Statement

Stratmann. Effect of Cold Atmospheric Plasma Therapy vs Standard Therapy Placebo on Wound Healing in Patients With Diabetic Foot Ulcers. *JAMA Netw Open*. Published July 16, 2020. 10.1001/jamanetworkopen.2020.10411

**Data**
**Data available:** No

**Additional Information**
**Explanation for why data not available:** Data will be available upon request following local data protection Rights (Germany)
